# Biomimetic and Bioactive Small Diameter Tubular Scaffolds for Vascular Tissue Engineering

**DOI:** 10.3390/biomimetics7040199

**Published:** 2022-11-14

**Authors:** Elisabetta Rosellini, Niccoletta Barbani, Luigi Lazzeri, Maria Grazia Cascone

**Affiliations:** Department of Civil and Industrial Engineering, University of Pisa, Largo Lucio Lazzarino, 56122 Pisa, Italy

**Keywords:** gelatin, gellan, elastin, endothelialization, functionalization, peptide

## Abstract

The present work aimed at the production and characterization of small caliber biomimetic and bioactive tubular scaffolds, which are able to favor the endothelialization process, and therefore potentially be suitable for vascular tissue engineering. The tubular scaffolds were produced using a specially designed mold, starting from a gelatin/gellan/elastin (GGE) blend, selected to mimic the composition of the extracellular matrix of native blood vessels. GGE scaffolds were obtained through freeze-drying and subsequent cross-linking. To obtain systems capable of promoting endothelization, the scaffolds were functionalized using two different bioactive peptides, Gly-Arg-Gly-Asp-Ser-Pro (GRGSDP) and Arg-Glu-Asp-Val (REDV). A complete physicochemical, mechanical, functional, and biological characterization of the developed scaffolds was performed. GGE scaffolds showed a good porosity, which could promote cell infiltration and proliferation and a dense external surface, which could avoid bleeding. Moreover, developed scaffolds showed good hydrophilicity, an elastic behavior similar to natural vessels, suitability for sterilization by an ISO accepted treatment, and an adequate suture retention strength. In vitro cell culture tests showed no cytotoxic activity against 3T3 fibroblasts. The functionalization with the REDV peptide favored the adhesion and growth of endothelial cells, while GRGDSP-modified scaffolds represented a better substrate for fibroblasts.

## 1. Introduction

Vascular diseases, including peripheral arterial occlusive diseases and coronary heart diseases, remain a leading cause of patient mortality and disability worldwide. Surgical repair is an indispensable treatment option in patients with advanced vascular disease, and vascular grafts are generally used to replace or bypass diseased vessels.

Synthetic vascular grafts have been found to be adequate in replacing large and medium caliber vessels. In contrast, small synthetic grafts have poor patency rates due to thrombus formation and intimal hyperplasia. Therefore, while having limitations, autologous vascular grafts are currently the only clinically viable options for the replacement of small caliber vessels [1].

An interesting alternative to traditional vascular prostheses implant is represented by vascular tissue engineering, whose purpose is to develop vascular grafts that integrate with the patient tissue and behave in a similar way to native vascular vessels.

The wall of native vessels is composed of three layers: an inner layer, called intima; a middle layer, called media; and an outer one, called adventitia. The intima is made up of connective tissue and a monolayer of endothelial cells (ECs), that lines the luminal surface of the wall in contact with the blood. The media consists of connective tissue (rich in collagen, elastin, and elastic fibers) and smooth muscle cells. The adventitia is made up of fibrous tissue, consisting of elastin and collagen fibers, and fibroblasts [2]. ECs of the intima play an important role in antithrombogenicity, by suppressing the activation of platelets and subsequent coagulation cascade [3,4]. Therefore, the formation of an endothelial layer on the inner surface of a TEVG is of fundamental importance to avoid thrombosis, which leads to stenosis and occlusion of the vascular lumen [5].

The materials selected to produce tissue engineered vascular grafts (TEVGs) must be biocompatible and not provoke an immune response. TEVGs should be able to mimic the native extracellular matrix (ECM), supporting the growth of cells and guiding tissue regeneration [4,6]. Moreover, they should exhibit long-term patency, an antithrombotic surface, and the ability to remodel in response to different stimuli [7].

Both natural and synthetic polymers have been used in literature to produce scaffolds for vascular tissue engineering [8]. A great advantage of synthetic polymers, such as biodegradable synthetic polyesters, is that their mechanical properties and degradation rate are adjustable [9]; however, they are unable to provide useful signals affecting cell adhesion and proliferation [10]. On the other hand, natural polymers present properties similar to those of the native ECM in terms of functional groups [8] and exhibit natural domains that are recognized by cells and able to influence their behavior. In numerous studies, mixtures of collagen and elastin have been used for the production of scaffolds, which are able to mimic the ECM composition of natural vessels [11,12,13,14]. However, collagen is potentially immunogenic due to its animal origin and it presents high costs. To overcome these limitations, an alternative could be represented by gelatin, which is obtained from collagen denaturation. Gelatin is biocompatible, biodegradable, non-immunogenic, easy to process, and inexpensive. Furthermore, it does not activate platelets, which is an important property for a material envisioned for vascular tissue engineering applications. Another limitation of collagen/elastin scaffolds is that they only reproduce the protein components of vascular ECM, which instead contains also glycosaminoglycans.

Therefore, the use of a blend based on natural polymers, of both protein and polysaccharide type, could represent a valid solution to produce a scaffold more closely mimicking the composition of the natural vascular ECM [15]. Moreover, the inclusion in the blend of a polysaccharide component could increase the number of functional groups available for subsequent scaffold functionalization.

Another crucial requirement in the development of a scaffold for vascular tissue engineering is in fact the ability to promote the formation of an endothelial layer on the inner surface, given that surface endothelialization is fundamental to guarantee the patency of the vessel.

Several strategies have been proposed in literature to promote endothelialization, inducing ECs migration, adhesion, and proliferation, or favoring the homing and directional differentiation of endothelial progenitor cells. 

Different surface modification methods, controlling scaffold microstructure [16,17,18,19,20] and physicochemical properties [21,22,23,24], have been investigated. Alternatively, endothelialization was promoted by loading the scaffold with biological factors, such as antibodies [25], growth factors [26], adhesive proteins [27], or short peptide sequences derived from them [28]. 

This last strategy is one of the most promising. Specific amino acid sequences, derived from ECM proteins, can mediate cell adhesion, proliferation, and differentiation by binding to integrins or other membrane protein receptors. The most widely investigated peptide sequence is the arginine-glycine-aspartic acid (RGD) sequence from fibronectin, which is recognized by integrin receptors that are present on the surface of all cell types [29]. Additionally, longer sequences are widely used for scaffold functionalization containing the RGD sequence, such as the sequence Gly-Arg-Gly-Asp-Ser-Pro (GRGDSP) derived from fibronectin, which has a demonstrated affinity toward integrin receptors that are present on the surface of various cells types, including ECs, fibroblasts, and smooth muscle cells [30,31]. Another peptide sequence proposed for the functionalization of scaffolds intended for the regeneration of blood vessels is the Arg-Glu-Asp-Val (REDV) sequence, from fibronectin. In contrast to the GRGDSP sequence, the REDV sequence specifically promotes the adhesion of ECs and endothelial progenitor cells. This sequence binds the αvβ3 integrin of ECs and several studies have shown that a highly uniform cell layer can be obtained [32,33].

In the present work, innovative scaffolds for the engineering of small diameter blood vessels have been developed. These scaffolds are based on the combination of: (i) biomimicry, given by the use as scaffold material of a protein/polysaccharide blend, mimicking native ECM composition; (ii) bioactivity, obtained by peptide coupling on scaffold surface, to induce endothelialization. 

Small diameter tubular scaffolds were produced, using a specially designed mold, starting from a polymer blend of gelatin, gellan, and elastin, through a freeze-drying process. 

Two different bioactive peptides were investigated for scaffold functionalization: the GRGSDP sequence, recognized by integrin receptors present on the surface of different cell types, and the REDV sequence, specifically recognized by integrin receptors present on the surface of ECs.

A complete characterization of the produced materials (GRGDSP- and REDV-modified scaffolds and unmodified scaffolds) was performed by carrying out morphological analysis, infrared analysis, thermal analysis, biomechanical characterization (including dynamic mechanical analysis, burst pressure strength, and suture retention strength), swelling and degradation tests. The suitability of the material to sterilization by an ISO accepted treatment was also verified. Finally, a biological in vitro characterization of the scaffolds was carried out, including cytotoxicity test with 3T3 fibroblasts and adhesion and proliferation tests with human umbilical vein endothelial cells (HUVEC) and human dermal fibroblasts (HDF).

## 2. Materials and Methods

### 2.1. Materials

Gelatin (type B from bovine skin), gellan, soluble elastin from bovine neck ligament, 1-(3-dimethylaminopropyl)-3-ethylcarbodiimide hydrochloride (EDC), N-hydroxysuccinimide (NHS), and phosphate buffered saline (PBS) were supplied by Sigma-Aldrich (Milan, Italy). The peptide sequences Gly-Gly-Gly-Arg-Glu-Asp-Val (GGGREDV) and Gly-Gly-Gly-Gly-Arg-Gly-Asp-Ser-Pro (GGGGRGDSP) were synthesized and provided by Cambridge Research Biochemicals (Billingham, Cleveland, UK). Moreover, calcium chloride and acetone were purchased from Carlo Erba Reagenti (Milan, Italy). All the other reagents were commercially available and used as received.

### 2.2. Mold Design 

To produce tubular scaffolds, an appropriate mold was designed. The mold was mechanized by the company Brusa srl, established in Livorno, Italy. 

**Figure 1:**
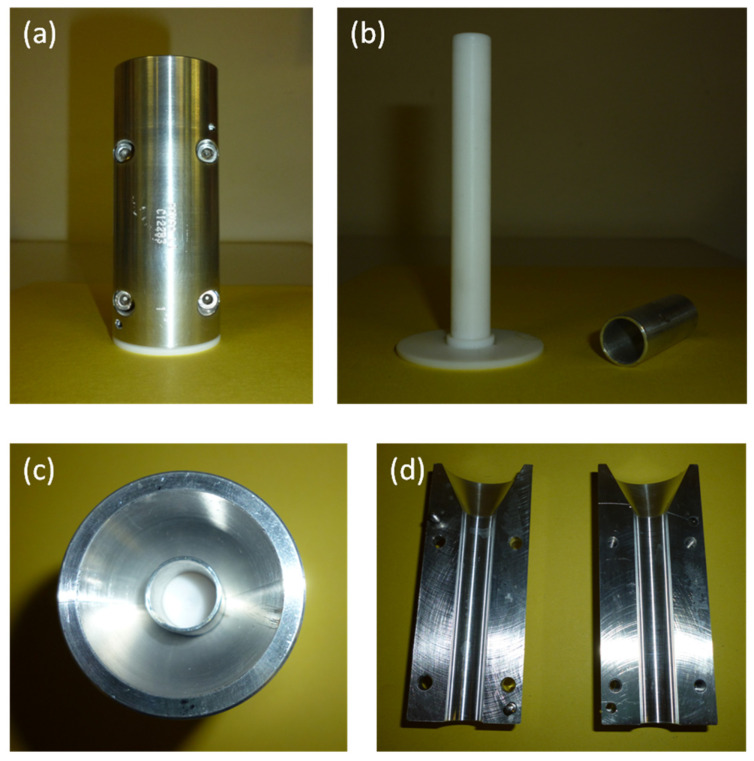
Mold designed for the production of tubular scaffolds by freeze-drying. (**a**) Complete mold; (**b**) internal mandrel and bush; (**c**) view from the top; (**d**) external chamber, open.

The mold was composed of two main parts: an internal mandrel and an external chamber, made by two semi-shells with four screws (Figure 1). 

The materials selected for mold fabrication were: Teflon for the internal mandrel, thanks to its chemical inertness, insolubility in water and organic solvents and excellent surface smoothness; aluminum for the external chamber, thanks to its high corrosion resistance and good temperature conductivity.

An aluminum bush was also used for maintaining the mandrel centered in the external chamber. 

The mold was designed to permit the production of tubular scaffolds with an internal diameter of 4 mm, a thickness of 1 mm, and a length of 7 cm.

### 2.3. Scaffold Fabrication Procedure

Biomimetic tubular scaffolds were produced by freeze-drying, starting from the preparation of a gelatin/gellan/elastin (GGE) blend. A 2% (*w*/*v*) GGE solution (with a weight ratio of 50/35/15 among the three components, respectively) was prepared in bi-distilled water at 50 °C. Then, the blend was introduced in the orifice of the blood vessel mold, frozen at −25 °C and lyophilized using a ΔT of 10 °C.

Since all the three biopolymers are soluble in water, scaffolds were cross-linked by treatment with EDC, for the stabilization of both protein components, and with a CaCl_2_ solution, for the ionic cross-linking of the polysaccharide component.

Preformed scaffolds were first immersed under stirring for 24 h at room temperature into an EDC/NHS solution in acetone/water = 90/10 (*v*/*v*), using an amount of EDC corresponding to 30% of protein content in the scaffold and a sufficient amount of NHS to have a 1/1 molar ratio between EDC and NHS. Then, samples were immersed, under stirring for 2 h at room temperature, in a 2% (*w*/*v*) CaCl_2_ solution. Thereafter, cross-linked scaffolds were washed three times with bi-distilled water, to remove unreacted compounds, and then freeze-dried.

### 2.4. Functionalization Procedure

As a further step toward the development of bioactive scaffolds, functionalization by immobilization of bioactive peptides was performed.

For this purpose, scaffolds were prepared into Petri dishes, rather than using the blood vessel mold.

EDC/NHS, used as cross-linking agents, are also coupling reagents which are able to activate the carboxylic groups contained in the blend, for subsequent amide bond formation with amine groups of bioactive peptides used for functionalization.

Both peptide sequences (GRGDSP and REDV) were synthesized by Cambridge Research Biochemicals (Billingham, Cleveland, UK), introducing a spacer, made by three residues of glycine, to increase their flexibility and thus favoring their interaction with the cell membrane integrin receptors. 

After the cross-linking process, scaffolds were introduced for 12 h, at 4 °C, in the coupling solution, made by GRGDSP or REDV peptide dissolved in PBS (pH = 7.4), at a concentration of 3.6 µmol/mL. 

Peptide-modified scaffolds were finally washed three times in bi-distilled water to remove unreacted compounds and avoid any potential cytotoxic effect, as already demonstrated in our previous paper in which surface coupling through EDC/NHS chemistry was investigated on different scaffold substrates [34,35]. Thoroughly rinsed scaffolds were then freeze-dried. GRGDSP- and REDV-modified scaffolds were subsequently compared with not functionalized ones.

### 2.5. Morphological Analysis

Morphological properties of the scaffolds, especially in terms of porosity (pores dimension and interconnection among them), were investigated by scanning electron microscopy, using the microscope JSM 5600 (Jeol Ltd., Tokyo, Japan). Before analysis, samples were mounted on metal stubs and sprayed with gold to a thickness of 200–500 Å using a gold splutter. Images were acquired at different magnifications, reported on the micrographs. The percentage of porosity was calculated by analyzing SEM images through the ImageJ software (version 1.53a, National Institutes of Health), as the ratio between pores area and total scaffold area.

### 2.6. Infrared Chemical Imaging Analysis

The scaffolds were subjected to infrared analysis to investigate: (i) the presence of interactions among components; (ii) the chemical homogeneity at the end of the fabrication process; (iii) the secondary structure of protein components; and (iv) the occurrence of the coupling reaction. 

A Fourier transformed infrared (FT-IR) spectrometer (Spectrum Spotlight 350 FT-NIR imaging system, Perkin Elmer, Waltham, MA, USA) was used.

To test the chemical homogeneity of the samples, spectral images were acquired using an infrared imaging system in the 4000–600 cm^−1^ range. From the chemical map, the medium spectrum (which is the most representative spectrum) was recorded. Then, using the instrument software, the correlation map was elaborated as the correlation among the chemical map and the medium spectrum. A correlation index close to one on all the investigated samples indicated the chemical homogeneity of the sample.

### 2.7. Thermal Analysis

The thermal behavior of the GGE scaffolds was investigated by a Perkin–Elmer DSC 7 differential scanning calorimeter (DSC), using aluminum pans. The scans were performed at a rate of 10 °C/min, from 20 to 250 °C.

### 2.8. Swelling Tests

The swelling properties of cross-linked scaffolds were evaluated both by exposure to aqueous vapor at 37 °C and by immersion in PBS, following standard procedures [15]. At appointed times, swelling percentage was evaluated according to the following equation:

(1) $$\mathrm{Swelling}\;\%=\frac{W_{s}-W_{d}}{W_{d}}\times 100$$

where *W_d_* is the starting dry weight and *W_s_* is the swollen weight. 

### 2.9. Degradation Tests

Degradation test was performed in agreement with the ISO norm 10993-13 “Biological evaluation of medical devices. Part 13: Identification and quantification of degradation products from polymeric medical devices” [36].

The in vitro hydrolytic degradation properties of cross-linked GGE scaffolds were evaluated in PBS (pH = 7.4). 

The weight loss of the scaffolds during degradation was determined by measuring changes in dry weight after specific incubation times. All experiments were performed in triplicate; the results are the mean (±SE) of three determinations carried out by keeping the samples in separate containers.

Test samples were prepared by cutting scaffolds into squares of 1 cm^2^. Samples were dried at room temperature to a constant mass and the starting dry weight, *W*_0_, was determined for each sample using a balance with adequate precision. Then, the samples were placed in containers, added with PBS in order to be completely immersed, closed, and placed in a stirring bath at 37 ± 1 °C for the entire duration of the degradation test.

At appointed times, samples were removed from the degradation solution using a weighted filter and rinsed in bi-distilled water. The filter and its content were dried at room temperature to a constant mass and the dry weight at time *t* of degradation, *W_t_*, was determined for each sample. Percentage weight loss was evaluated according to the following equation:

(2) $$\mathrm{Weight}\;\mathrm{loss}\;\%=\frac{W_{0}-W_{t}}{W_{0}}\times 100$$

In addition to the determination of percentage weight loss during degradation, degraded samples were characterized by infrared analysis, to understand the mechanism of hydrolysis.

### 2.10. Biomechanical Characterization

#### 2.10.1. Dynamic Mechanical Analysis (DMA)

The viscoelastic properties of GGE scaffolds were investigated using a dynamic mechanical analyzer (DMA8000, Perkin-Elmer, Waltham, MA, USA), following the protocol described in our previous paper [15]. Before analysis, samples were equilibrated for 1 h in PBS, at 37 °C, and tests were carried out under wet conditions. Single strain (10 µm)/multi-frequency (1, 3.5, and 10 Hz) tests were performed and storage modulus (E’), loss modulus (E’’), and tangent delta (tan δ) were determined.

#### 2.10.2. Burst Pressure Strength

The burst pressure for the tubular scaffolds was measured by increasing the pressure of a fluid within them, until failure or leakage occurred. A 9% (*w*/*v*) aqueous solution of polyvinylpyrrolidone (PVP) was used, being the viscosity of this solution similar to blood [37]. A peristaltic pump was introduced to test the burst pressure strength. The pressure was gradually increased until failure or leakage occurred and pressure change was recorded through a digital manometer. Triplicate samples were tested and average values were obtained.

#### 2.10.3. Suture Retention Strength

The suture retention strength of the scaffolds was measured according to the guidelines of the American National Standard Institute for the Advancement of Medical Instruments 7198, 2016 [38]. Rectangular samples of 20 × 10 mm were prepared. Samples were immersed in PBS for 1 h prior to testing to simulate the hydrated condition at the implant site. One end of the sample was fixed to the stage clamp of the Instron tensile tester (Instron 5542 dynamometer, Instron, Turin, Italy). The opposite end was connected to another clamp of the testing device by polypropylene suture, creating a single loop, 2 mm from the short edge. 

The tests were carried out at room temperature by setting a deformation speed of 50 mm/min to pull the suture and using a load cell of 50 N. The samples were subjected to a longitudinal tension force.

The maximum load prior to pull-through of the suture was recorded as the suture retention strength.

### 2.11. Determination of Peptide Surface Density

High performance liquid chromatography (HPLC, 200 Series HPLC system, Perkin Elmer, with a UV/VIS detector) was used to determine the peptide surface density, by measuring the corresponding concentration in the coupling solution after coupling reaction (*C_f_*), as well as in the wash waters (*C_w_*). An HP Prosphere C4 300A 5u column (250 mm length × 4.5 mm internal diameter, Alltech Associates, Deerfield, IL) was used. The mobile phase was: *A* = 0.085% trifluoroacetic acid (*w*/*v*) in acetonitrile; B = 0.1% trifluoroacetic acid (*w*/*v*) in water. The elution condition was a linear binary gradient at a flow rate of 1 mL/min and the gradient was from 30% A and 70% B to 60% A and 40% B in 15 min. The injection volume was 50 µL. The detector wavelength was set at λ = 280 nm. Knowing the concentration of the coupling solution before the reaction (*C_i_*), the volume of the coupling solution and of the wash waters (*V*) and the area (*A*) of the treated surface, the peptide surface density was calculated according to the following equation:

(3) $$\mathrm{Density}=\frac{V\left(C_{i}-C_{f}-C_{w}\right)}{A}$$

### 2.12. Suitability for Sterilization

GGE samples were sterilized by Gamma irradiation, which is a sterilization procedure accepted by the ISO norm [39]. To investigate the suitability of the material to the sterilization process, infrared and mechanical analysis were carried out on the sterilized samples, according to the procedures already described under Section 2.6 and Section 2.10.1. Results were compared with those obtained before sterilization.

### 2.13. In Vitro Biological Characterization

#### 2.13.1. Cytotoxicity Tests

GGE scaffolds were tested for cytotoxicity using a 3T3 embryonic mouse fibroblast cell line (European Collection of Cell Culture, London, UK), according to the ISO norm 10993-5 “Biological evaluation of medical devices—Test for cytotoxicity: in vitro methods” [40]. The cytotoxicity test was performed by direct contact; 3T3 fibroblasts (about 10^6^ cells/mL) suspended in DMEM containing 10% (*v*/*v*) fetal bovine serum (FBS), 2 mM glutamine, penicillin (100 U/mL), and streptomycin (100 µg/mL), were seeded in 24-well plates, using 1 mL for each well. After 24 h of seeding, the medium was replaced with fresh medium and a sterilized unmodified GGE sample was placed in the center of each well. Polystyrene (culture dish surface) was used as the negative control, while 2,4-dinitrophenol (70 μg/mL) was added to the positive control wells. Cell culture was carried out for 72 h inside an incubator at 37 °C in a humidified atmosphere containing 5% CO_2_. After 72 h of incubation, cell viability was measured by (3-(4,5-dimethylthiazol-2-yl)-2,5-diphenyltetrazolium bromide (MTT) assay as follows. MTT solution was added to the wells in an amount equal to 10% (*v*/*v*) of the culture volume, followed by incubation at 37 °C for 4 h. At the end of the incubation time, the resulting formazan precipitate was dissolved by the addition of MTT solvent in an amount equal to the original culture volume. An UV spectrophotometer (Shimadzu, UV-2100) was used to measure the absorbance at 570 nm.

#### 2.13.2. Cell Adhesion and Proliferation Tests

Both HUVEC and HDF were used to perform cell adhesion and proliferation tests. HUVEC were used to study the capability of peptide-modified scaffolds to promote endothelialization; HDF were used to investigate the effect on a different cell type which is present in native vessels. REDV-modified GGE scaffolds, GRGDSP-modified GGE scaffolds and unmodified GGE scaffolds (n = 3 for each sample type) were first sterilized by the following protocol: washing in water/ethanol with 70% ethanol, drying under a sterile hood, and UV exposure for 15 min on each side. Prior to cell seeding, the scaffolds were placed in 96-well plates, washed 3 times with sterile 2x Pen-Strep (#P0781, Sigma-Aldrich, Milan, Italy)/Diflucan (Pfizer, Rome, Italy) saline, and rinsed with PBS. HUVEC and HDF were seeded on the scaffolds at a density of 2 × 10^5^ cells/sample in a volume of 15 µL of complete culture medium. Endothelial Cell Growth Medium (ECGM, #211-500, Cell application inc, Sand Diego, CA, US) was used for HUVEC and Dulbecco’s Modified Eagle Medium (DMEM, #11054-020, Gibco, Milan, Italy) for HDF, both added with fetal bovine serum (10%), 2 mM glutamine, penicillin (100 U/mL), and streptomycin (100 µg/mL). The plates were placed for 1 h in the incubator, for cell adhesion, then complete medium was added and the cells were cultured for 11 days, providing media changes every 3 days.

The Alamar Blue assay (#BUF012A, Serotec Ltd., Oxford, UK) was used to test the viability and proliferation of cells seeded onto the GGE samples. This bioassay uses a redox indicator, which causes the culture media to change color in response to cell metabolism. The low toxicity of this assay allows it to be repeated numerous times on the same samples. In accordance with the manufacturer’s instructions, samples and controls—including scaffolds devoid of cells used as blank controls—were incubated with the dye for 3 h. To determine cell viability, samples were analyzed at three different culture times: 3, 7, and 11 days after seeding. At the appointed times, the culture supernatants were taken out and fresh culture media was added. A spectrophotometer (Victor3, PerkinElmer, Waltham, MA, USA) was used to analyze supernatants under a double wavelength reading of 570 and 600 nm. Finally, the manufacturer’s recommended absorbance calculations and dye molar extinction coefficients were used to obtain the dye reduction percentage.

#### 2.13.3. Statistical Analysis

Statistical significance in Alamar Blue assay was evaluated using the 2-tailed *t*-test for paired data, followed by Bonferroni’s correction.

## 3. Results and Discussion

### 3.1. Morphological Analysis

SEM micrographs of GGE tubular scaffolds were acquired before and after cross-linking.

**Figure 2:**
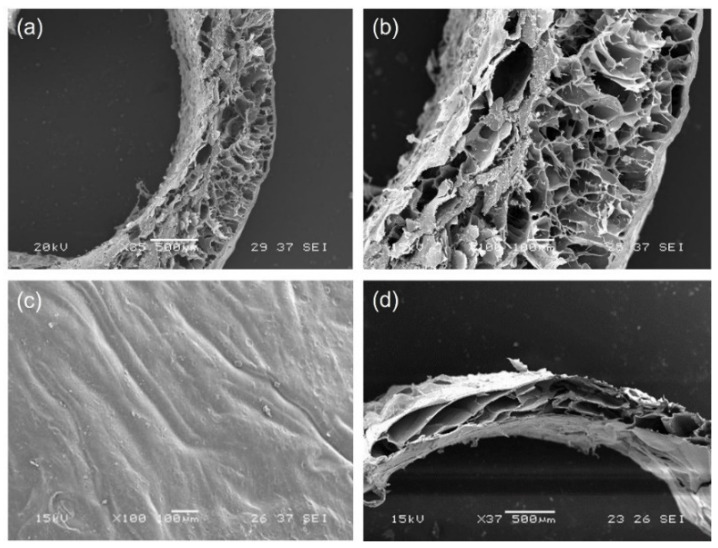
SEM micrographs of: (**a**,**b**) section and (**c**) external surface of untreated GGE tubular scaffold; (**d**) section of cross-linked GGE tubular scaffold.

The morphological analysis of untreated scaffolds (Figure 2a–c) showed the presence of a good porosity in sample section, which could promote cell infiltration and proliferation. Pore diameter increased from the external surface (where the average pore diameter was around 100 µm) to the internal lumen (where the average pore diameter was around 250 µm); the wall thickness of dried samples was 800 µm. The external surface appeared dense, which is a desired property to avoid bleeding. 

After cross-linking, we observed a contraction of scaffold material, with a decrease in wall thickness of dried scaffolds to 600 µm (Figure 2d). However, the section of the scaffold maintained a high degree of porosity. 

The percentage of porosity, as calculated by ImageJ analysis of SEM images, was 43%.

SEM micrographs of tubular scaffolds prepared in different times were also compared to evaluate the reproducibility of the preparation procedure (see Supplementary Figure S1). The images acquired showed only small differences in pore dimensions, imputable to the intrinsic variability of the freeze-drying process.

### 3.2. Infrared Chemical Imaging Analysis

**Figure 3:**
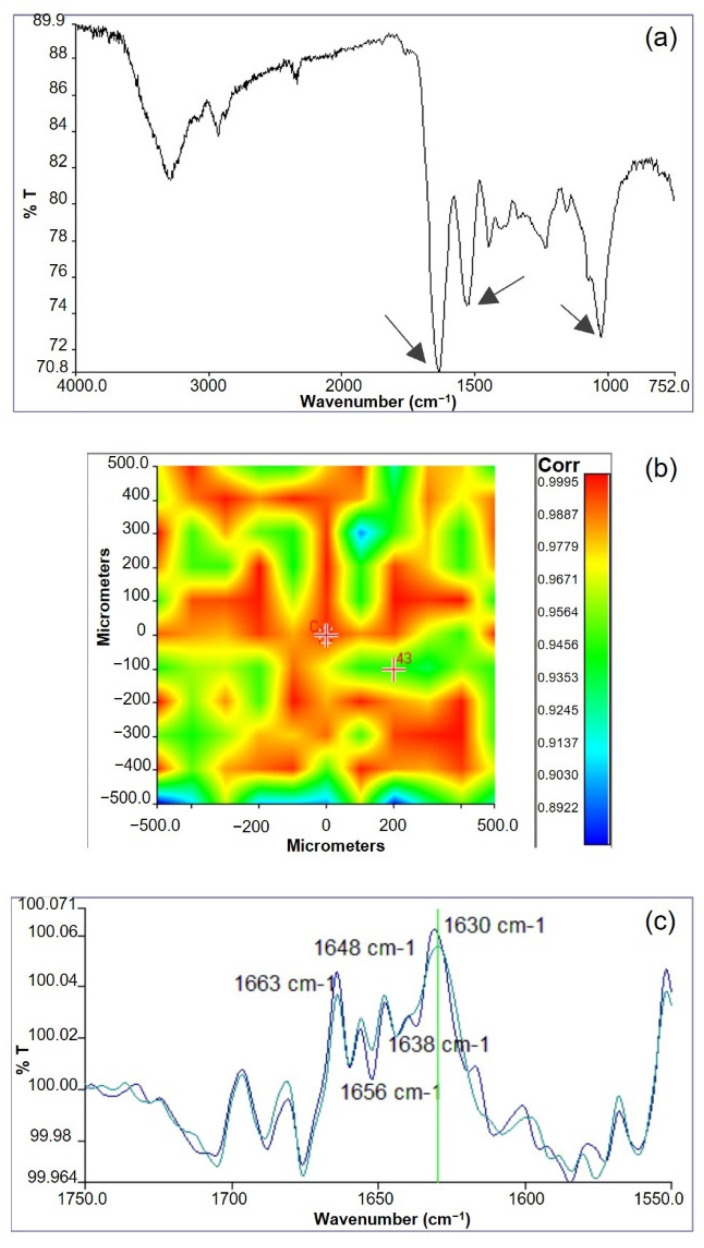
Infrared analysis of GGE scaffolds: (**a**) Medium spectrum recorded from the chemical map; (**b**) correlation map between the chemical map and the medium spectrum; (**c**) second derivative spectra.

Infrared chemical imaging analysis was carried out on the scaffolds. The chemical map was acquired and the medium spectrum, which is the most representative of the chemical map, was recorded. The medium spectrum (Figure 3a) was compared with those of pure polymers (see Supplementary Figure S2). The two proteins are characterized by absorption bands at 1650 cm^−1^, due to the stretching of C=O group in Amide I, and at 1538 cm^−1^, due to the N-H bending in Amide II. Gellan is characterized by an absorption band at 1024 cm^−1^, due to the C-O-C group of the polysaccharide ring. The FT-IR medium spectrum of the scaffold showed the presence of all the absorption peaks typical of both protein and polysaccharide components, even if band displacements were observed. In particular, the Amide II band moved to 1550 cm^−1^ and the C-O-C band moved to 1035 cm^−1^. These band displacements were explained by the establishment of interactions among the protein and the polysaccharide components [41]. It was supposed that these interactions are due to the formation of hydrogen bonds between the N-H group of the proteins and glycosidic group of gellan, as already observed in similar protein/polysaccharide blends [15,42].

The correlation map between the chemical map and the medium spectrum was elaborated to investigate sample homogeneity. The correlation map (Figure 3b) revealed correlation values close to 1 on all the analyzed sample, demonstrating the high chemical homogeneity of the scaffold. The high chemical homogeneity can be considered a consequence of the interactions among the protein and the polysaccharide components, demonstrated by the displacement of the adsorption bands due to Am II and C-O-C of polysaccharide ring, as previously discussed. 

Furthermore, the protein secondary structure was investigated by acquiring second derivative spectra (Figure 3c). It is well known that the deconvolution of Amide I band is relevant for the identification of polypeptide conformation [43]. Second derivative spectra of GGE scaffolds showed the presence of typical peaks, due to β-sheet (1663 cm^−1^), α-helix (1650 cm^−1^), β-unordered (1630 cm^−1^), and triple helix (1638 cm^−1^) structures. In particular, the presence of this last band suggested a partial reorganization of protein material, probably due to the interactions established among blend components [44]. However, the triple helix band was less evident than in other protein/polysaccharide blends [15,42]. This could be explained by milder interactions between components in the GGE blend developed in this work, with respect to alginate/gelatin [15] and alginate/gelatin/elastin [42] blends investigated in previous works.

**Figure 4:**
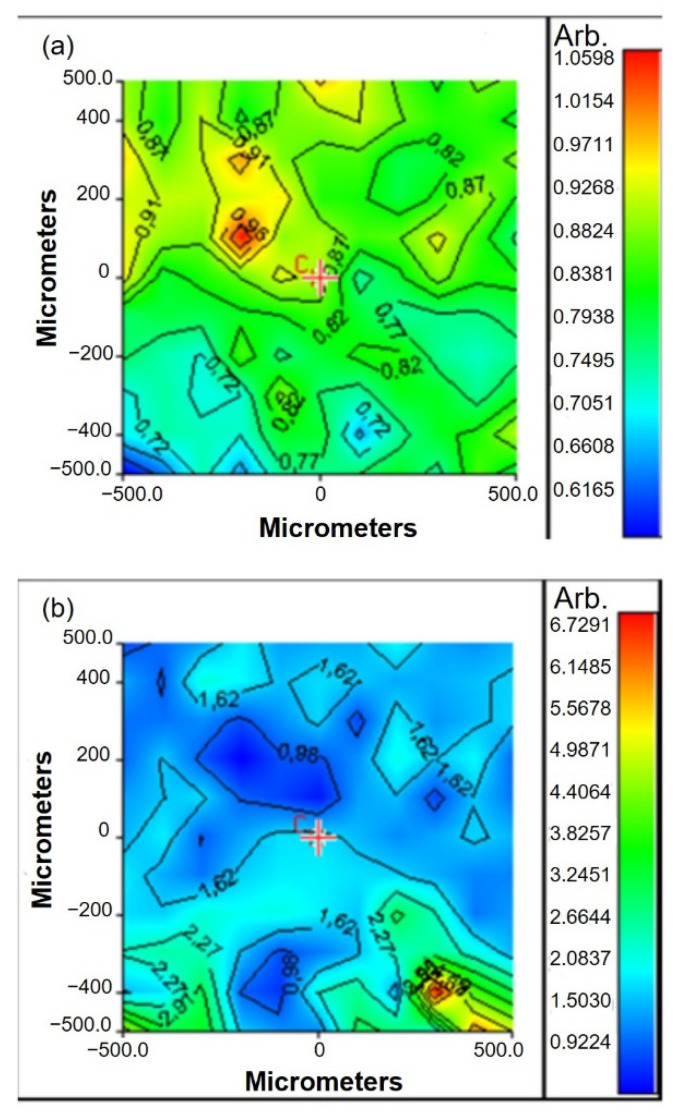
Chemical maps, in function of Am II/C-O-C band ratio, acquired: (**a**) before functionalization; (**b**) after functionalization.

Chemical imaging analysis was also performed after GGE scaffold functionalization (Figure 4). The band ratio between Amide II absorption peak (of protein material) and C-O-C absorption peak (of gellan) was used to verify the functionalization of scaffold surfaces with peptides. Chemical maps, in function of Am II/C-O-C band ratio, were acquired before and after functionalization. 

The value of band ratio before functionalization was in the interval 0.6–1 (Figure 4a). After functionalization, a significant increase in the band ratio was observed, which could be explained by the increase in protein content after peptide binding (Figure 4b).

### 3.3. Thermal Analysis

Thermal analysis was carried out by DSC to investigate the presence of interactions between blend components [41]. 

**Figure 5:**
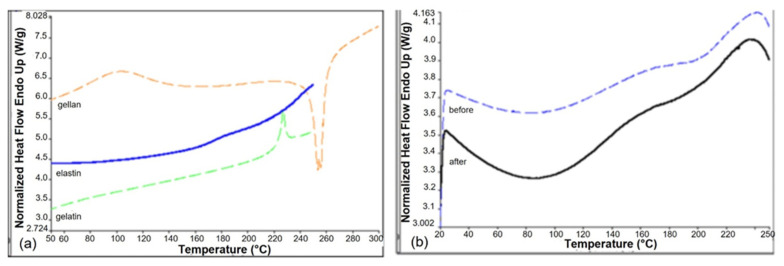
DSC thermograms of pure components (**a**) and of GGE tubular scaffolds, before and after cross-linking (**b**).

Thermograms of GGE scaffolds (Figure 5b), before and after cross-linking, were compared with those of pure components (Figure 5a). Gellan thermogram showed a first endothermic event between 50 and 130 °C, due to the evaporation of residual water, and a second exothermic event at 260 °C, due to the polysaccharide degradation. Elastin thermogram showed a variation of the baseline, between 160 and 190 °C, attributable to the glass transition event. Gelatin showed a glass transition event at 220 °C, superimposed to an endothermic event, due to the denaturation of residual triple helix.

In the thermogram of the GGE blend we observed only one thermal event, between 130 and 190 °C, which can be attributed to the glass transition of the protein components. The presence in the blend of only one glass transition event could be explained by the establishment of strong interactions between gelatin and elastin. 

The thermogram of cross-linked scaffolds was identical to non-cross-linked ones, suggesting that the cross-linking process did not alter the interactions between components.

### 3.4. Swelling Tests

Swelling tests were carried out to investigate the ability of the material to absorb water. This property is related to the degree of hydrophilicity and depends on the chemical characteristics of blend components, the degree of cross-linking, the interactions between the components, and the morphological properties of the produced materials. 

**Figure 6:**
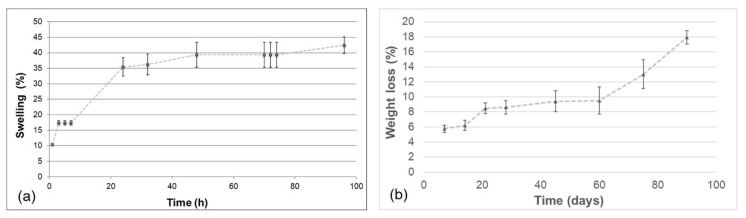
Swelling kinetic for cross-linked GGE scaffold exposed to water vapours (**a**) and weight loss kinetic during the hydrolysis of cross-linked GGE scaffolds (**b**).

GGE cross-linked samples were first exposed to water vapors and the percentage of swelling was calculated using the formula reported in Section 2.8, obtaining the trend over time, as represented in Figure 6a. The swelling percentage of absorbed water increased, until it reached the equilibrium swelling value of 42%.

The obtained value of swelling degree was only slightly lower than that obtained in a previous work [42] for alginate/gelatin/elastin scaffolds and this difference can be explained by the different polysaccharides used (gellan rather than alginate) and by the different weight ratios between components. 

Swelling tests were also performed by immersion in PBS solution at 37 °C. The cross-linked GGE sample reached an equilibrium swelling percentage of 145 ± 7%, only after 1 h of immersion. 

Overall, these results demonstrated a good hydrophilicity of the prepared scaffolds.

### 3.5. Degradation Tests

Degradation tests were performed maintaining the cross-linked GGE samples in PBS, at 37 °C, into an agitating bath. At appointed times, the percentage of weight loss was evaluated.

The weight loss kinetic showed a rapid release of material during the first week; then the sample continued to release material more slowly up to 60 days, when a recovery of the hydrolysis process was observed (Figure 6b). After 90 days, 80% of scaffold material was still present.

Infrared analysis of degraded samples was carried out. Spectra of degraded materials showed the same absorption peaks of non-degraded samples. However, a decrease in the intensity for the absorption band at 1050 cm^−1^ (due to gellan) was observed. Therefore, to verify whether the weight loss during hydrolysis was mainly due to the release of the polysaccharide component, the band ratio between the absorption peaks of the protein materials (Am I + Am II, between 1727 and 1485 cm^−1^) and the absorption peak of the polysaccharide (C-O-C, between 1181 and 945 cm^−1^) was calculated. An increase in the band ratio was found during hydrolysis, from 1.22 before hydrolysis, to 1.43 after one month of hydrolysis and 1.69 after 3 months of hydrolysis. These results suggested that the weight loss during hydrolysis was mainly due to gellan release.

### 3.6. Biomechanical Characterization

#### 3.6.1. Dynamic Mechanical Analysis (DMA)

In Table 1, the results of the mechanical analysis, performed by DMA, are shown. Values of storage modulus (E’), loss modulus (E’’), and tan delta were determined at three different frequencies (1, 3.5, and 10 Hz), corresponding to healthy human heart rate, pathological human heart rate, and a fatigue condition with a supraphysiological pulse rate, respectively [15]. 

Values of E’ were one order of magnitude higher than values of E’’, with E’ on the order of 10^4^ Pa, over the entire frequency range, indicating the elastic behavior of the developed scaffolds [45].

As expected, increasing the oscillation frequency, an increase was observed for values of E’ and E’’. This result demonstrated that the stiffness of GGE scaffolds increased with frequency increase, as typically occurs for hydrogel scaffolds [46]. This stiffening involves both an increase in elasticity (E’) and an increase in heat dissipation (E’’). 

Values of tan δ were around 0.1 and did not show variations by increasing frequency, suggesting that E’ and E’’ increased with frequency at the same proportion. 

DMA was used in literature to evaluate the viscoelastic properties of blood vessels and their substitutes [47]. The storage modulus measured for GGE scaffold in this work is lower than reported by Khosravi et al. for autologous graft (1.55 MPa for small saphenous vein and 5.79 MPa for internal thoracic artery [48]). However, native vessels showed a similar elastic behavior (with E’ around an order of magnitude above E’’) and values of tan delta close to 0.1 [48], as found in this work for GGE scaffolds. Moreover, the viscoelastic characterization by DMA of scaffolds for vascular tissue engineering application carried out by Liu et al. [49] reported values of storage modulus on the order of 10^4^ Pa and values of loss modulus on the order of 10^3^ Pa, similarly to what is obtained in this work.

#### 3.6.2. Burst Pressure Strength

The burst pressure strength is one of the most important properties for blood vessel scaffolds. To evaluate whether GGE tubular scaffolds had sufficient strength to withstand physiological forces, burst pressure tests were performed to determine the maximum pressure that GGE vascular scaffolds could endure before failure. Six GGE tubular scaffolds were used and a burst pressure strength of 150 ± 27 mmHg was measured. 

As the normal blood pressure in the human body is 90–120 mmHg [50] and the ideal burst pressure for blood vessel scaffolds is 2000 mmHg [51], the value measured for GGE scaffolds can be considered sufficient but not optimal.

To improve this property, we tested the dipping of the developed scaffolds in a gellan solution. Gellan was dissolved in bi-distilled water, at a concentration of 2% (*w*/*v*). GGE scaffolds were mounted on the mandrel of the mold, immersed in the gellan solution for 5 min, and then dried. This procedure was carried out three times. Burst pressure tests were repeated after dipping, showing a significant increase in the burst pressure strength up to 320 ± 23 mmHg, which is a value closer to what is reported in literature for other blood vessel scaffolds based only on natural polymers [47].

#### 3.6.3. Suture Retention Strength

The ability of a scaffold to withstand forces due to suturing is a crucial parameter in the fabrication of scaffold for blood vessel tissue engineering, as it determines the success of the graft implantation procedure. Experiments carried out in this work showed a suture retention strength of 2.2 ± 0.1 N for hydrated GGE scaffolds. Reference values reported in literature show a suture retention strength of 1.96 N for human arteries [52]. Therefore, the obtained results suggest that the suture retention strength of GGE scaffolds was more than adequate for suturing during implantation.

### 3.7. Determination of Peptide Surface Density

The peptide surface density on functionalized scaffold was determined by quantifying, through HPLC analysis, the amount of residual peptide in the coupling solution after the coupling process.

Considering the molecular weight of the peptide sequences, the immobilized peptide density resulted in 0.17 ± 0.02 µmol/cm^2^ for scaffolds modified with GRGDSP and 0.24 ± 0.03 µmol/cm^2^ for scaffolds modified with REDV. It is well known in literature that there is a relationship between cell response and the surface concentration of immobilized peptides [53]. In particular, surface modification with GRGDSP and REDV peptides has been investigated in literature on materials different from GGE scaffold [32,54]. According to the results reported in these studies, a peptide density on the order of pmol/cm^2^ is sufficient to promote cell adhesion and proliferation. Although cell attachment is influenced not only by peptide density, but also by other parameters, such as the surface properties of the material used as substrate [55], the peptide density obtained on functionalized GGE scaffolds is several orders of magnitude above these reference values. Therefore, it should be adequate to affect cell response.

### 3.8. Suitability for Sterilization

Checking suitability for sterilization is of paramount importance in the characterization of scaffolds for tissue engineering applications. Materials intended to come into contact with biological tissues must necessarily be sterilized to avoid contamination, through an ISO norm-approved sterilization method, and the sterilization procedure must not change their properties. 

The sterilization of GGE scaffolds was performed by Gamma irradiation, a procedure that is in agreement with the ISO norms on sterilization of medical devices [39]. The suitability for sterilization of the prepared scaffolds was investigated by comparing the physicochemical and mechanical characteristics of the materials, after the sterilization process, with those observed before sterilization. 

The infrared and mechanical tests performed on the sterilized scaffolds did not reveal any significant variation with respect to the results obtained for non-sterilized samples (see Supplementary Figure S3 and Supplementary Table S1), demonstrating that the polymeric scaffolds developed in this work can be sterilized by ISO recommended treatments.

### 3.9. In Vitro Biological Characterization

#### 3.9.1. Cytotoxicity Tests

In vitro cytotoxicity tests were performed according to ISO norm 10993-5. Cytotoxicity was tested by direct contact, using murine fibroblast 3T3. Cell viability was measured 72 h after seeding by MTT assay, and reading absorbance at 570 nm. 

The results were expressed in terms of percentage of cell viability, which was calculated as follows:

(4) $$\mathrm{Cell}\;\mathrm{viability}\;\%=\frac{\mathrm{Absorbance}\;\left(570\;\mathrm{nm}\right)\;\mathrm{test}\;\mathrm{product}}{\mathrm{Absorbance}\;\left(570\;\mathrm{nm}\right)\;\mathrm{negative}\;\mathrm{control}}\times 100$$

Reduction in cell viability by more than 50% with respect to negative control was considered a cytotoxic effect.

**Figure 7:**
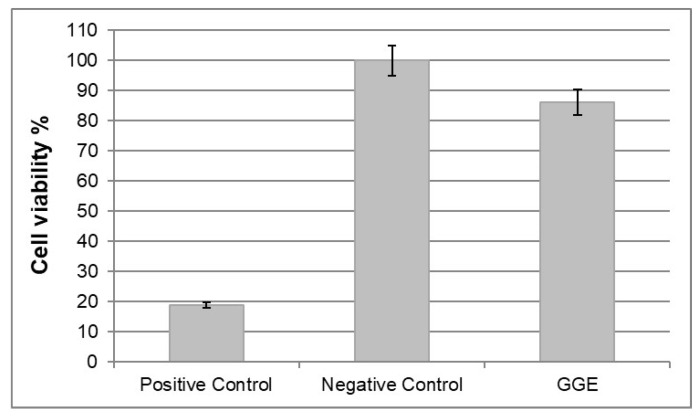
Results of cytotoxicity test on GGE scaffolds.

The results are collected in Figure 7. No cytotoxic activity against murine fibroblast 3T3 was detected for GGE unmodified scaffolds. 

#### 3.9.2. Cell Adhesion and Proliferation Tests

**Figure 8:**
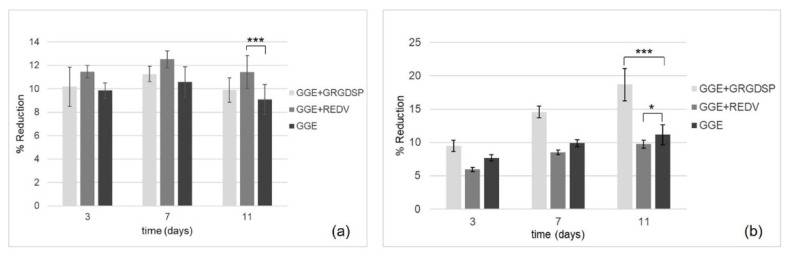
Alamar Blue reduction percentage as a function of culture times for scaffolds seeded with HUVEC (**a**) and HDF (**b**). GRGDSP-modified scaffolds (GGE+GRGDSP) and REDV-modified scaffolds (GGE+REDV) were compared with unmodified scaffolds (GGE). The data (n = 9; error bars ± SD) were compared using Student’s *t*-test and differences were considered significant when * *p* < 0.05, *** *p* < 0.001.

The in vitro Alamar Blue assay was performed to evaluate the viability and proliferation of HUVEC and HDF seeded onto the scaffolds. Samples were analyzed at three different culture times, 3, 7, and 11 days after seeding. The spectrophotometric analysis of culture supernatants was performed under a double wavelength reading of 570 and 600 nm. The results, expressed in terms of percentage of dye reduction, showed that the functionalization of the scaffolds promoted cell adhesion and proliferation. With regard to HUVEC (Figure 8a), the highest percentage of reduction (corresponding to a higher cell viability and therefore to a higher number of cells on the scaffold) was observed for the REDV-modified samples. On the other hand, with respect to HDF (Figure 8b), the highest percentage of reduction was observed for the GRGDSP-modified samples. 

In both cases, an increase in the percentage of reduction was observed as a function of time, suggesting the ability of the cells which are adhered to the materials to proliferate.

## 4. Conclusions

Novel biomimetic and bioactive small diameter tubular scaffolds were produced by freeze-drying and subsequent cross-linking, starting from a polymer blend of gelatin, gellan, and elastin, which are able to mimic the protein/polysaccharide components that are present in the ECM of native vessels. 

The scaffolds were functionalized using two different bioactive peptides: GRGSDP, recognized by integrin receptors present on the surface of different cell types, and REDV, specifically recognized by integrin receptors present on the surface of ECs. 

A complete physicochemical, mechanical, functional, and biological characterization of the produced materials (GRGDSP- and REDV-modified scaffolds and unmodified scaffolds) was performed. 

The produced GGE scaffolds showed a good porosity, which could promote cell infiltration and proliferation, and a dense external surface that could avoid bleeding. The infrared chemical imaging analysis pointed out a high chemical homogeneity of the material and the establishment of interactions between components, which produced a partial reorganization of protein material. In addition, this analysis confirmed the occurrence of the coupling reaction with the peptide sequences. Peptide surface density was quantified by HPLC analysis and resulted higher than reference values reported in literature to promote a biological response. The GGE scaffolds showed good hydrophilicity, and suitability for sterilization by an ISO accepted treatment. The material showed an elastic behavior similar to natural vessels. The measured burst pressure strength of 150 mmHg was well below the ideal value of 2000 mmHg. However, it was demonstrated that it can be easily increased above physiological and pathological pressure values, by simply dipping the scaffold in a gellan solution. Suture retention strength resulted adequate to withstand a surgical implantation procedure. 

No cytotoxic activity against 3T3 murine fibroblast was detected for GGE unmodified scaffolds. Cell adhesion and proliferation tests were carried out with HUVEC and HDF, comparing peptide-modified scaffolds with unmodified ones. The functionalization of the scaffolds promoted cell adhesion and proliferation. The best substrates for HUVEC were the GGE scaffolds functionalized with the REDV peptide. In the case of samples seeded with HDF, the best results were obtained with scaffolds functionalized with GRGDSP, while samples functionalized with REDV behave worse than unmodified control. An increase in the number of cells was also observed as a function of time, indicating the ability of adhered cells to proliferate.

Overall, the obtained results look promising and suggest a potential use of the developed GGE scaffolds for in vitro vascular tissue engineering, while their application for in situ strategies would request a further improvement in mechanical properties, especially in terms of burst pressure strength. Results of biological characterization indicate that functionalization of the GGE scaffolds with the REDV peptide favors the adhesion and growth of ECs and therefore could be advantageous for the endothelialization of the internal surface of an engineered vessel, while that with GRGDSP promotes the adhesion of fibroblasts, which are present in the external layer of blood vessel. Therefore, as a further step toward the development of a biomimetic and bioactive scaffold for vascular tissue engineering, a bi-functionalized tubular scaffold could be developed. REDV could be grafted on the luminal side of the scaffold, to promote specifically endothelial cell adhesion, while GRGDSP could be used for the functionalization of the external side, to promote fibroblasts and smooth muscle cells adhesion. This bi-functionalized scaffold could be able to promote the regeneration of a layered tissue, resembling the native structure of small-caliber arteries.

## Figures and Tables

**Table 1:** Storage modulus (E’), loss modulus (E’’), and tan delta of GGE scaffolds, measured by DMA at three different frequencies (1, 3.5, and 10 Hz).

	E’ (Pa)	E’’ (Pa)	Tan δ
1 Hz	(5.30 ± 0.07) × 10^4^	(5.10 ± 0.17) × 10^3^	0.10 ± 0.02
3.5 Hz	(5.49 ± 0.08) × 10^4^	(5.52 ± 0.09) × 10^3^	0.10 ± 0.01
10 Hz	(5.69 ± 0.11) × 10^4^	(5.63 ± 0.07) × 10^3^	0.10 ± 0.01

## Data Availability

The data presented in this study are available within the article.

## Supplementary Materials

The following supporting information can be downloaded at: https://www.mdpi.com/article/10.3390/biomimetics7040199/s1. Figure S1: SEM micrographs of three different prototypes; Figure S2: Comparison between the infrared spectrum of GGE (in black) and the infrared spectrum of pure components, gelatin (in red) and gellan (in blue); Figure S3: Infrared spectra of GGE scaffolds, before and after sterilization by Gamma irradiation; Table S1: Storage modulus (E’), loss modulus (E’’), and tan delta of GGE scaffolds, measured by DMA at three different frequencies (1, 3.5, and 10 Hz), after scaffold sterilization by Gamma irradiation.
