# Supplementary material for: Biomimetic and Bioactive Small Diameter Tubular Scaffolds for Vascular Tissue Engineering
Source: Biomimetics (Basel). 2022 Nov 14;7(4):199. doi: 10.3390/biomimetics7040199 (PMC9680506; doi:10.3390/biomimetics7040199)
Supplement: Supplementary file 1 [file biomimetics-07-00199-s001.zip › SupplemetaryTableS1.pdf]

Table S1. Storage modulus (E′), loss modulus (E′′) and tan delta of GGE scaffolds, measured by DMA at three different frequencies (1, 3.5 and 10 Hz), after scaffold sterilization by gamma irradiation.

	**E′ (Pa)**	**E′′ (Pa)**	**Tan δ**
1 Hz	$(5.21 \pm 0.15) \times 10^4$	$(5.18 \pm 0.06) \times 10^3$	0.10 ± 0.02
3.5 Hz	$(5.38 \pm 0.04) \times 10^4$	$(5.46 \pm 0.09) \times 10^3$	0.10 ± 0.01
10 Hz	$(5.73 \pm 0.07) \times 10^4$	$(5.70 \pm 0.04) \times 10^3$	0.10 ± 0.01
