# Supplementary figures and images for: Biomimetic and Bioactive Small Diameter Tubular Scaffolds for Vascular Tissue Engineering
Source: Biomimetics (Basel). 2022 Nov 14;7(4):199. doi: 10.3390/biomimetics7040199 (PMC9680506; doi:10.3390/biomimetics7040199)

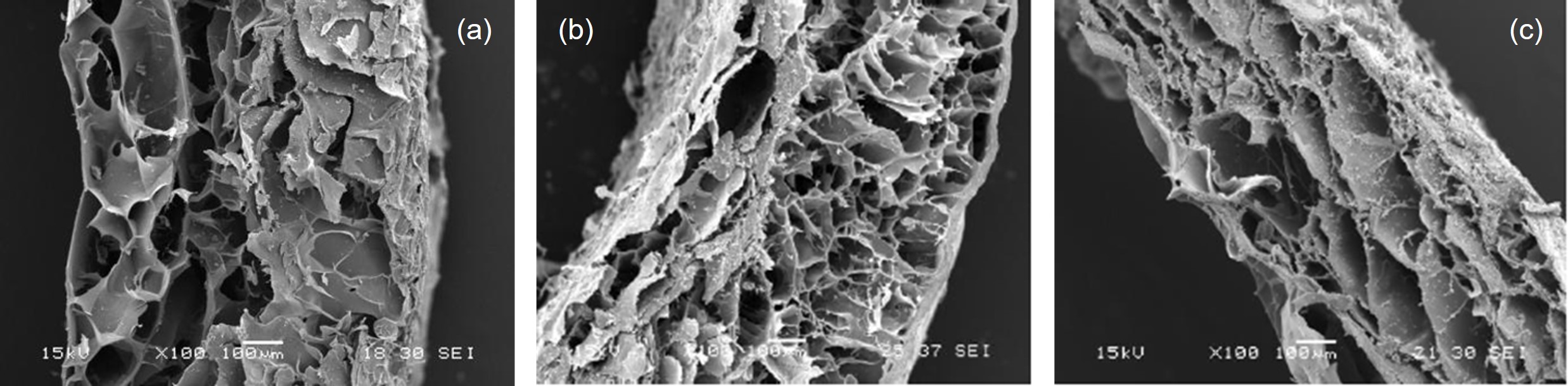

Supplement: Supplementary file 1 [file biomimetics-07-00199-s001.zip › FigureS1_RoselliniE.jpg]

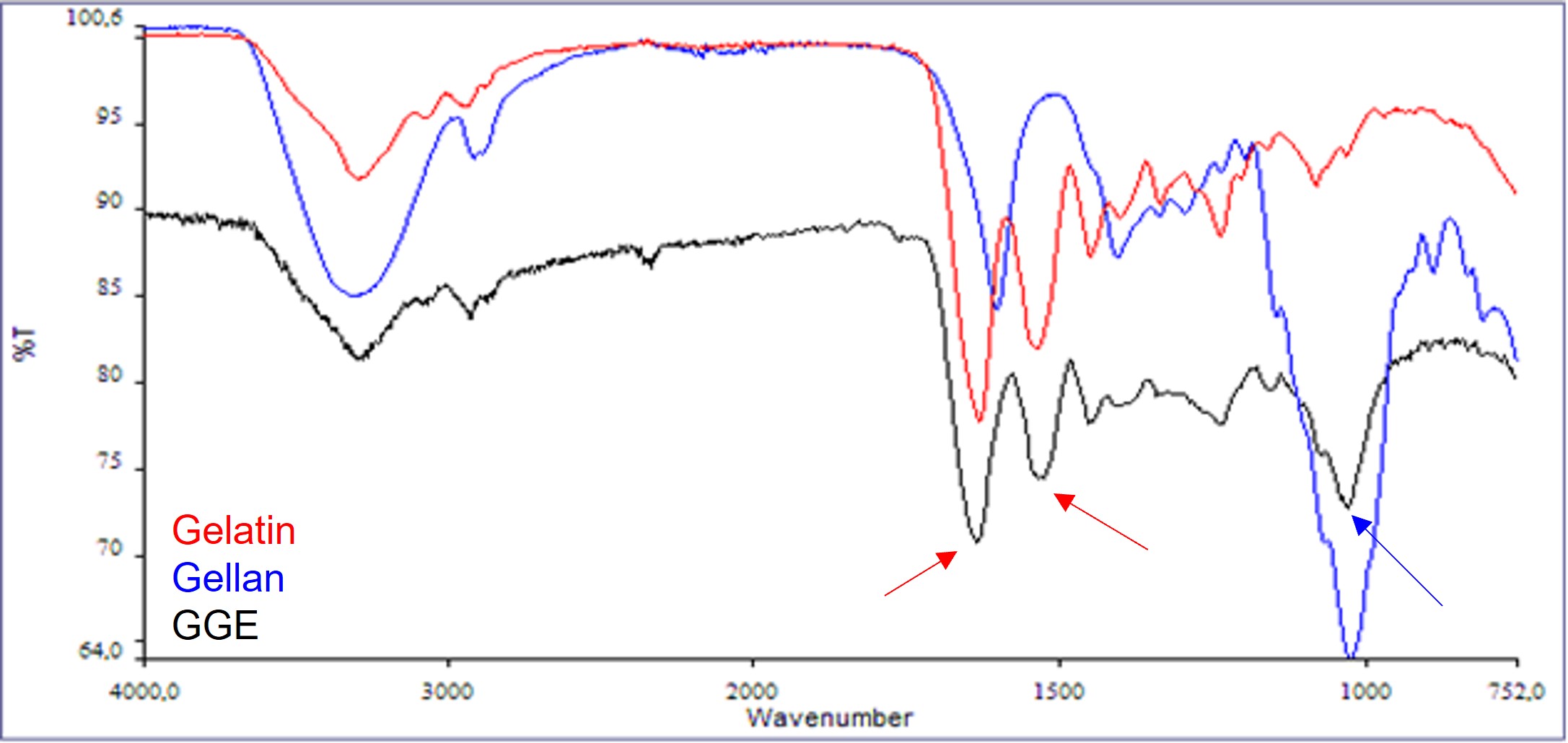

Supplement: Supplementary file 1 [file biomimetics-07-00199-s001.zip › FigureS2_RoselliniE.jpg]

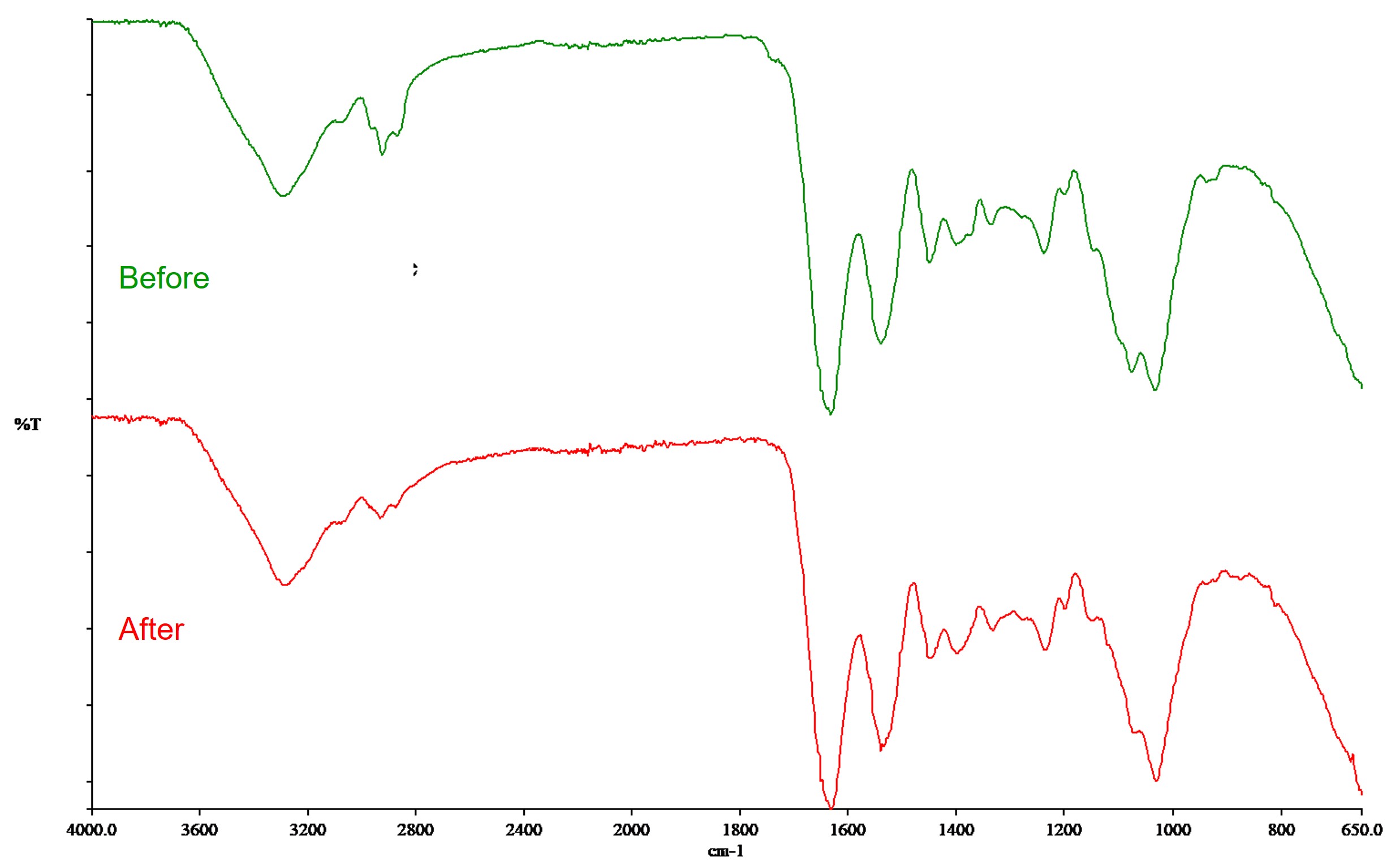

Supplement: Supplementary file 1 [file biomimetics-07-00199-s001.zip › FigureS3_RoselliniE.jpg]
